# Genomic Network-Based Analysis Reveals Pancreatic Adenocarcinoma Up-Regulating Factor-Related Prognostic Markers in Cervical Carcinoma

**DOI:** 10.3389/fonc.2018.00465

**Published:** 2018-10-23

**Authors:** Jihye Kim, Joon-Yong Chung, Tae-Joong Kim, Jeong-Won Lee, Byoung-Gie Kim, Duk-Soo Bae, Chel Hun Choi, Stephen M. Hewitt

**Affiliations:** ^1^Departments of Obstetrics and Gynecology, Samsung Medical Center, Sungkyunkwan University School of Medicine, Seoul, South Korea; ^2^Experimental Pathology Laboratory, Laboratory of Pathology, Center for Cancer Research, National Cancer Institute, National Institutes of Health, Bethesda, MD, United States

**Keywords:** PAUF, AGR2, BRD7, POM121, prognosis, uterine cervical neoplasms

## Abstract

We previously showed that PAUF is involved in tumor development and metastases in cervical cancer. This study was conducted to discover novel molecular markers linked with PAUF in cervical cancer using genomic network analysis and to assess their prognostic value in cervical cancer. Three PAUF-related genes were identified using *in-silico* network-based analysis of the open genome datasets. To assess the expression of these genes and their relationship to the outcome of cervical cancer, immunohistochemical analysis was performed using cervical cancer TMA. The associations of the identified proteins with clinicopathologic characteristics and prognosis were examined. AGR2, BRD7, and POM121 were identified as interconnected with PAUF through *in-silico* network-based analysis. AGR2 (*r* = 0.213, *p* < 0.001) and POM121 (*r* = 0.135, *p* = 0.013) protein expression were positively correlated with PAUF. BRD7^High^ and AGR2^Low^ were significantly associated with favorable disease-free survival (DFS) (*p* = 0.009 and *p* < 0.001, respectively), and in combination with PAUF^High^, even more significantly favorable DFS observed (*p* < 0.001 for both). In multivariate analysis, AGR2^High^ (HR = 3.16, *p* = 0.01) and BRD7^High^ (HR = 0.5, *p* = 0.025) showed independent prognostic value for DFS. In a random survival forest (RSF) model, the combined clinical and molecular variable model predicted DFS with significantly improved power compared with that of the clinical variable model (C-index of 0.79 vs. 0.75, *p* < 0.001). In conclusion, AGR2 and BRD7 expression have prognostic significance in cervical cancer and provide opportunities for improved treatment options. Genomic network-based approaches using the cBioPortal may facilitate the discovery of additional biomarkers for the prognosis of cervical cancer and may provide new insights into the biology of cervical carcinogenesis.

## Introduction

Cervical cancer is the second most commonly diagnosed cancer and is the third leading cause of cancer death among females in less-developed countries (1). Although screening for precancerous lesions and human papilloma virus (HPV) infection and preventive HPV vaccinations are good options to prevent cervical cancer, once invasive cancer occurs, recurrence remains a major problem despite improvements in treatment (2).

To improve survival in cervical cancer patient, proper treatment should be adopted. If tumor behavior could be reliably predicted at the initial diagnosis, tailored treatments could be implemented to improve survival. Although clinical factors such as International Federation of Gynecology and Obstetrics (FIGO) stage, lymph node metastasis, and tumor size may serve as markers for overall prognosis, they have limitations in accurately predicting survival (3). Thus, novel biomarkers including molecular markers are needed for accurate survival estimates. Recent advances in molecular biology and well-curated, publicly available data, such as The Cancer Genome Atlas (TCGA), have made it possible to identify multiple valuable prognostic biomarkers for cancer studies.

We previously reported that pancreatic adenocarcinoma up-regulated factor (PAUF) can be used as a prognostic molecular marker in patients with cervical cancer (4). PAUF expression is upregulated in cancers, particularly in glandular cells or adenocarcinoma, and cytoplasmic expression is independently associated with poor survival. However, the specific receptor involved, its downstream signaling pathways, and the molecular mechanisms of PAUF-dependent gene regulation need to be elucidated. Recently, there have been several network-based studies utilizing interaction information between genes in cancer (5, 6). Considering the complex nature of the interaction between genes, a single genetic abnormality can spread along the links of the complex intracellular network to alter the functions of related genes (7–9). In addition, we hypothesized that multi-gene markers could have more accuracy in prognostication than a single molecular marker (10). To this end, the markers interconnected with PAUF could be identified with *in silico* analysis using publicly available datasets. The purpose of this study was to find novel proteins connected with PAUF in cervical cancer using *in silico* analysis and to investigate the clinical significance of those proteins in a well-defined cohort of cervical cancers using immunohistochemistry.

## Materials and methods

### *in silico* analysis to select candidate genes connected with PAUF

**Figure 1:**
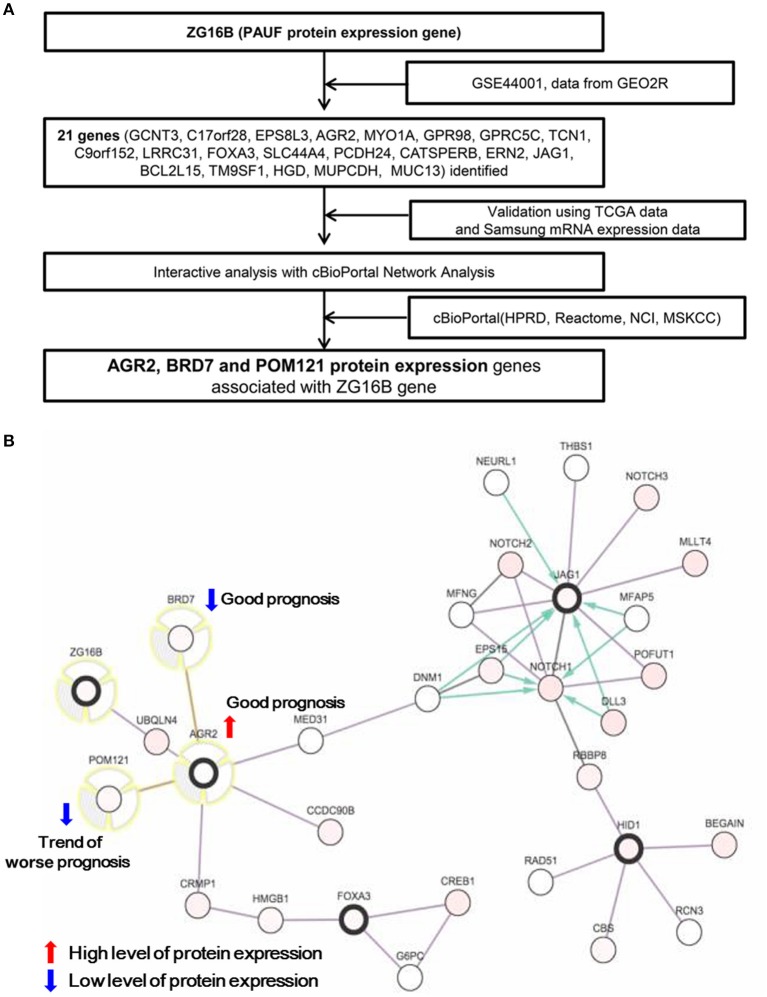
**(A)** Flow chart identifying the candidate PAUF-associated prognostic markers. **(B)** Network view of 22 genes using interactive analysis with cBioPortal network analysis (http://cbioportal.org). Among candidate genes, high expression of AGR2 protein and low expression of BRD7 protein are significantly associated with favorable disease-free survival while the low expression of POM121 protein shows a trend of worse prognosis in patients with cervical cancer.

To identify the novel prognostic molecular markers related to PAUF protein expression, data from the Gene Expression Omnibus (GSE44001) and TCGA datasets were analyzed, and a detailed schematic is depicted in Figure 1A. For the details, we downloaded the GES44001 dataset from GEO website (https://www.ncbi.nlm.nih.gov/geo/query/acc.cgi?acc=GSE44001). The obtained data were dichotomized by the median expression of ZG16b (Entrez Gene ID: 124220, ID: ILMN_1753139), and each 150 samples were allocated as high or low PAUF expression (File S1). Subsequently, the differential expression analysis was performed with the “Analyze with GEO2R” in the GEO website with options of Benjamini & Hochberg (False discovery rate), Auto-detect, and Submitter supplied (https://www.ncbi.nlm.nih.gov/geo/query/acc.cgi?acc=GSE44001). The detailed instruction of GEO2R is available in https://www.ncbi.nlm.nih.gov/geo/info/geo2r.html#how_to_use. The genes selected after the above steps are described in Table S1. An interactive analysis was conducted to explore genes related to ZG16B, the PAUF protein expression gene, using mutual exclusivity and network analysis with cBioPortal network analysis. cBioPortal for Cancer Genomics (http://cbioportal.org) is a web resource for exploring, visualizing, and analyzing multi-dimensional cancer genomics data (11). The detailed queries for the analysis are as follows; samples were “cervical squamous cell carcinoma and endocervical adenocarcinoma,” and genomic profile was “mRNA Expression z-Scores (RNA Seq V2 RSEM)”. The access date was November 2014.

### Patients and tumor samples

As previously described, tissue microarrays (TMAs) were constructed using samples from 336 early-stage cervical cancer patients who were primarily treated with radical hysterectomy at the Department of Gynecologic Oncology, Samsung Medical Center, between 2002 and 2009 (12, 13). Tissue samples were collected from patients who had signed an informed consent form and the study was approved by the Institutional Review Board at Samsung Medical Center, Seoul, Korea (IRB No: 2015-07-122). No patients had undergone previous radiation or chemotherapy. All patients underwent radical hysterectomy with pelvic lymphadenectomy. Para aortic lymph node sampling was performed on patients who were diagnosed with pelvic lymph node metastasis in frozen biopsy during operation, or patients with para aortic lymph node metastasis in pre-operative radiologic findings. Patients with rare histology or limited availability of tissue block specimens were excluded from the TMA cohort. Following radical hysterectomy, adjuvant radiotherapy with or without concurrent chemotherapy was given according to the following risk factors: lymph node metastasis, parametrial involvement, positive resection margins, stromal invasion of more than half of the cervix, lymphovascular space invasion (LVSI), or a tumor larger than 4 cm in diameter. During follow-ups conducted every 3 months for 2 years then every 6 months for the next 3 years, Pap smears, tumor markers, and CT scans were performed every 3–12 months. Patients who relapsed within 3 years after adjuvant chemoradiation were classified as resistant to chemoradiation. Disease-free survival (DFS) was assessed from the date of surgery to the date of recurrence or the date of the last follow-up visit. Overall survival (OS) was measured from the date of surgery to the time of death or the date of last contact (for living patients). The data for patients who had not experienced an event as of the date of the final observation were censored.

### Immunohistochemistry

Immunohistochemistry was performed using cervical cancer TMAs as previously described (12, 13). TMA sections were cut at 4 μm thickness followed by deparaffinization in xylene and dehydration with graded ethanols. The deparaffinized and rehydrated slides were treated with heat-induced antigen retrieval buffer (Dako, Carpinteria, CA) at pH 6.0 (for BRD7 and POM121) or pH 9.0 (for AGR2) for 20 min. The endogenous peroxidase activity was blocked with 3% H_2_O_2_ for 10 min. The sections were incubated with anti-AGR2 rabbit polyclonal antibody (Novus Biologicals, NBP2-27393, CO, USA) at a 1:250 dilution, anti-BRD7 rabbit polyclonal antibody (Novus Biologicals, NBP1-28727, CO, USA) at a 1:500 dilution, or anti-POM121 rabbit polyclonal antibody (Novus Biologicals) at a 1:500 dilution for 120 min at room temperature. The antigen-antibody reaction was detected with EnVision+ Dual Link System-HRP (Dako) and DAB+ (3,3′-Diaminobenzidine; Dako). Negative controls were performed by omitting the primary antibody and rabbit immunoglobulin (IgG).

### Quantitative evaluation of immunohistochemical staining

Immunohistochemical staining was quantitatively evaluated using computer-assisted image analysis software (Visiopharm, Hoersholm, Denmark) as described previously (14). In brief, the slides were scanned using a whole-slide scanner (NanoZoomer 2.0, Hamamatsu Photonics, Hamamatsu City, Japan) and imported into Visiopharm software (for Windows 7, version 4.5.1.324) using the TMA workflow. Staining intensity was categorized as 0, 1+, 2+, or 3+ according to the distribution pattern across cores. A brown staining intensity (0-negative, 1-weak, 2-moderate, or 3-strong) was obtained using the predefined algorithm and optimized settings. The overall immunohistochemical scores (histoscore) for AGR2, BRD7, and POM121 were expressed as the percentage of positive cells multiplied by their staining intensity (possible range, 0–300). For the survival analysis and hierarchical clustering, the expression values were dichotomized (High vs. Low, described in superscript) using cut-off values affording the most discriminative power (histoscore of 39 for AGR2, 46 for BRD7, and 204 for POM121). PAUF protein expression was evaluated as previously described (4) to compare with that of the three proteins identified.

### Statistical analysis

Statistical analysis was performed using R software, version 3.1.3 (R Foundation, Vienna, Austria; http://www.R-project.org). The expression levels of the proteins according to the clinicopathological characteristics were analyzed using Student's *t*-test or Mann–Whitney *U*-test. Analysis using Spearman's rho coefficient was used to assess the correlations between the expressions of the proteins. Survival distributions were estimated using the Kaplan–Meier method, and the relationship between survival and each parameter was analyzed with the log-rank test. A Cox proportional hazards model was created to identify independent predictors of survival. Statistical significance was defined as *p* < 0.05. Hierarchical clustering was performed to determine the clustering of samples according to PAUF and PAUF-related protein expression. In addition, the random survival forest (RSF) method was modified to include both clinical and molecular features to assess the predictive power of integrating the molecular data with clinical variables (15). A detailed description of the analysis has been previously described (12).

## Results

### Candidate genes connected with PAUF (ZG16B)

Analysis of the GSE44001 dataset showed that 21 genes were differentially expressed according to *ZG16B* gene expression (dichotomized using the median cutoff). These identified genes were validated in the TCGA cervix dataset (Table S1). The cBioPortal network view of these genes showed that anterior gradient protein 2 (AGR2), bromodomain-containing protein 7 (BRD7), and nuclear envelope pore membrane protein (POM121) are interconnected with the *ZG16B* gene (Figure 1B). These three proteins were selected to evaluate their prognostic value in cervical carcinoma.

### Clinicopathological characteristics of patients

The clinicopathological characteristics are summarized in Table 1. The mean age of the patients was 48.9 ± 11.2 years. Of the 336 patients with cervical cancer, 291 (86.6%) were stage IIA or less, and 45 (13.4%) were stage IB2 or IIB. In Korea, many surgeons perform radical hysterectomy on patients who were suspected of having a stage IIB diagnosis in pre-operative radiologic findings or physical examination. In this study, we intended to include possible samples which were obtained from radical hysterectomy even if the sample sizes were small. Of the patients, 256 (76.2%) had squamous cell carcinoma and 80 (23.8%) had adenocarcinoma. Lymph node metastasis was found in 80 (23.8%) patients and parametrial involvement in 31 (9.2%) patients. One hundred and sixty (47.6%) patients were treated with adjuvant radiation with or without concurrent chemotherapy due to the presence of risk factors. Among them, 20 patients were radiation resistant.

**Table 1:** The correlation between the PAUF associated proteins expressions with clinicopathologic characteristics of cervical cancer.

	**No**.	**AGR2**		**BRD7**		**POM121**	
		**Mean Histoscore [95% CI]**	***p-*****value**	**Mean Histoscore [95% CI]**	***p-*****value**	**Mean Histoscore [95% CI]**	***p-*****value**
**GROUP**							
Normal	81	92 [85–99]	0.724	266 [256–275]	<0.001^*^	228 [209–247]	<0.001^*^
Cancer	336	90 [81–99]		63 [59–68]		144 [134–153]	
**AGE**							
<50 years	203	94 [83–105]	0.274	61 [55–67]	0.227	143 [131–155]	0.902
>50 years	133	84 [70–98]		67 [59–74]		145 [128–161]	
**FIGO STAGE**							
≤IIA	291	88 [79–98]	0.346	64 [59–68]	0.749	141 [131–152]	0.208
IB2 or IIB	45	101 [76–126]		61 [50–73]		158 [134–183]	
**CELL TYPE**							
SCC	256	70 [62–79]	<0.001^*^	67 [62–72]	0.004^*^	148 [137–159]	0.123
AC/ASC	80	154 [136–172]		52 [44–60]		130 [111–150]	
**TUMOR SIZE**							
<4 cm	256	93 [83–103]	0.267	64 [59–69]	0.64	143 [132–154]	0.764
>4 cm	80	81 [64–99]		61 [52–71]		146 [127–165]	
**LVSI**							
Negative	202	99 [88–111]	0.007^*^	63 [57–69]	0.792	149 [136–162]	0.192
Positive	133	76 [63–89]		64 [57–71]		136 [122–150]	
**DEPTH OF INVASION**							
<50%	108	103 [87–119]	0.042^*^	57 [48–65]	0.047^*^	140 [123–158]	0.645
>50%	228	84 [73–94]		66 [61–72]		145 [134–157]	
**LN METASTASIS**							
Negative	256	92 [82–102]	0.438	63 [57–68]	0.664	144 [133–155]	0.901
Positive	80	84 [67–101]		65 [56–73]		143 [123–162]	
**PM INVOLVEMENT**							
Negative	305	92 [82–101]	0.218	62 [57–67]	0.082	145 [135–155]	0.291
Positive	31	74 [47–101]		75 [61–89]		130 [102–157]	
**RESECTION MARGIN**							
Negative	323	91 [82–100]	0.446	63 [59–68]	0.765	144 [134–154]	0.957
Positive	13	72 [19–124]		67 [41–92]		142 [82–203]	
**RT RESISTANCE**							
Resistant	20	102 [71–134]	0.212	45 [28–62]	0.053	89 [62–116]	0.001^*^
Sensitive	113	81 [66–95]		63 [56–70]		147 [130–164]	
**PRIMARY TREATMENT**							
OP only	171	97 [84–110]	0.266	65 [59–72]	0.704	143 [129–157]	0.989
OP+ RT	70	75 [58–92]		63 [52–74]		145 [123–167]	
OP + CCRT	90	88 [72–104]		60 [52–67]		144 [127–162]	
Neoadjuvant	5	87 [35–139]		53 [-9–114]		131 [-7–268]	

FIGO, International Federation of Gynecology and Obstetrics; SCC, squamous cell carcinoma; AC, adenocarcinoma; ASC, adenosquamous cell carcinoma; LVSI, lymphovascular space invasion; LN, lymph node; PM, parametrium; OP, operation; RT, radiotherapy; CCRT, concurrent chemoradiation.

* *Significant at the level of p < 0.05*.

### AGR2, BRD7, and POM121 protein expression

**Figure 2:**
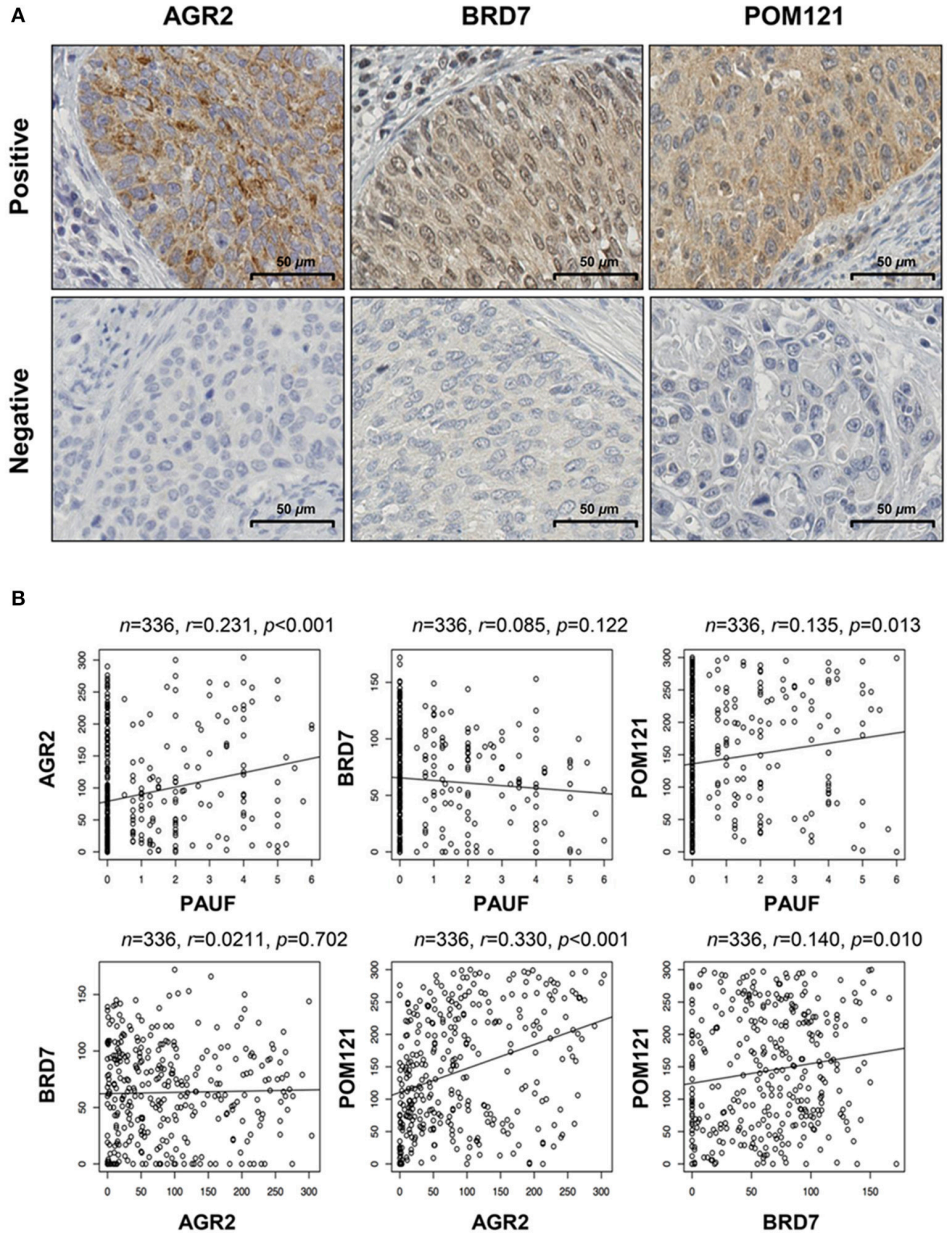
Expression of the PAUF-associated proteins AGR2, BRD7, and POM121 in patients with cervical cancer. **(A)** Representative examples of positive and negative expression of the AGR2, BRD7, and POM121 proteins. The scale bar represents 50 μm. **(B)** Correlations between expression of PAUF and PAUF-related proteins in cervical cancer tissues. AGR2 protein expression was positively correlated with POM121 expression (*r* = 0.33, *p* < 0.001).

AGR2 and POM121 protein expression was mainly observed in the cytoplasm, and BRD7 expression was observed in both the cytoplasm and nucleus. Representative examples of positive and negative staining are shown in Figure 2A. Subsequently, we examined the correlations between PAUF protein expression and these three proteins using subgroup analysis. Although there were no statistically significant differences, AGR2 and POM121 protein expression showed the tendency of a positive association with PAUF protein expression (*r* = 0.213; *p* < 0.001 and *r* = 0.135; *p* = 0.013, respectively; Figure 2B). Moreover, AGR2 protein expression was positively associated with POM121 protein expression as shown in Figure 2B (*r* = 0.330; *p* < 0.001). In the subgroup analysis, PAUF and POM121 protein expression was positively correlated with AGR2 protein expression (*r* = 0.305; *p* = 0.001 and *r* = 0.456; *p* < 0.001, respectively) in the radiation-sensitive group (*n* = 113; Figure S1A). Furthermore, in the radiation-resistant group (*n* = 20), BRD7 protein expression was positively associated with POM121 protein expression (*r* = 0.55; *p* = 0.012) (Figure S1B). These findings are consistent with the TCGA dataset and suggest that these proteins are correlated in cervical cancer, especially within subgroups according to radiation therapy.

When compared with normal cervix tissues, a significant decrease in BRD7 (mean histoscore 63 vs. 266, *p* < 0.001) and POM121 (mean histoscore 144 vs. 228, *p* < 0.001) expression was detected in cancer tissues, but the expression of AGR2 showed no significant difference (Table 1). The expression of AGR2 and BRD7 was cell type-dependent; AGR2 was more highly expressed in adeno/adenosquamous carcinoma, and BRD 7 expression was more prominent in squamous cell carcinoma (*p* < 0.001 and *p* = 0.004, respectively; Table 1). These results suggest that AGR2 and BRD7 potentially have different roles in cervical cancer according to cell type. In addition, the higher expression of AGR2 was negatively correlated with LVSI and the depth of invasion (*p* = 0.007 and *p* = 0.042, respectively), and POM121 expression was decreased (*p* = 0.001) in radiation-resistant cervical cancer.

### Unsupervised hierarchical clustering of PAUF-associated markers

**Figure 3:**
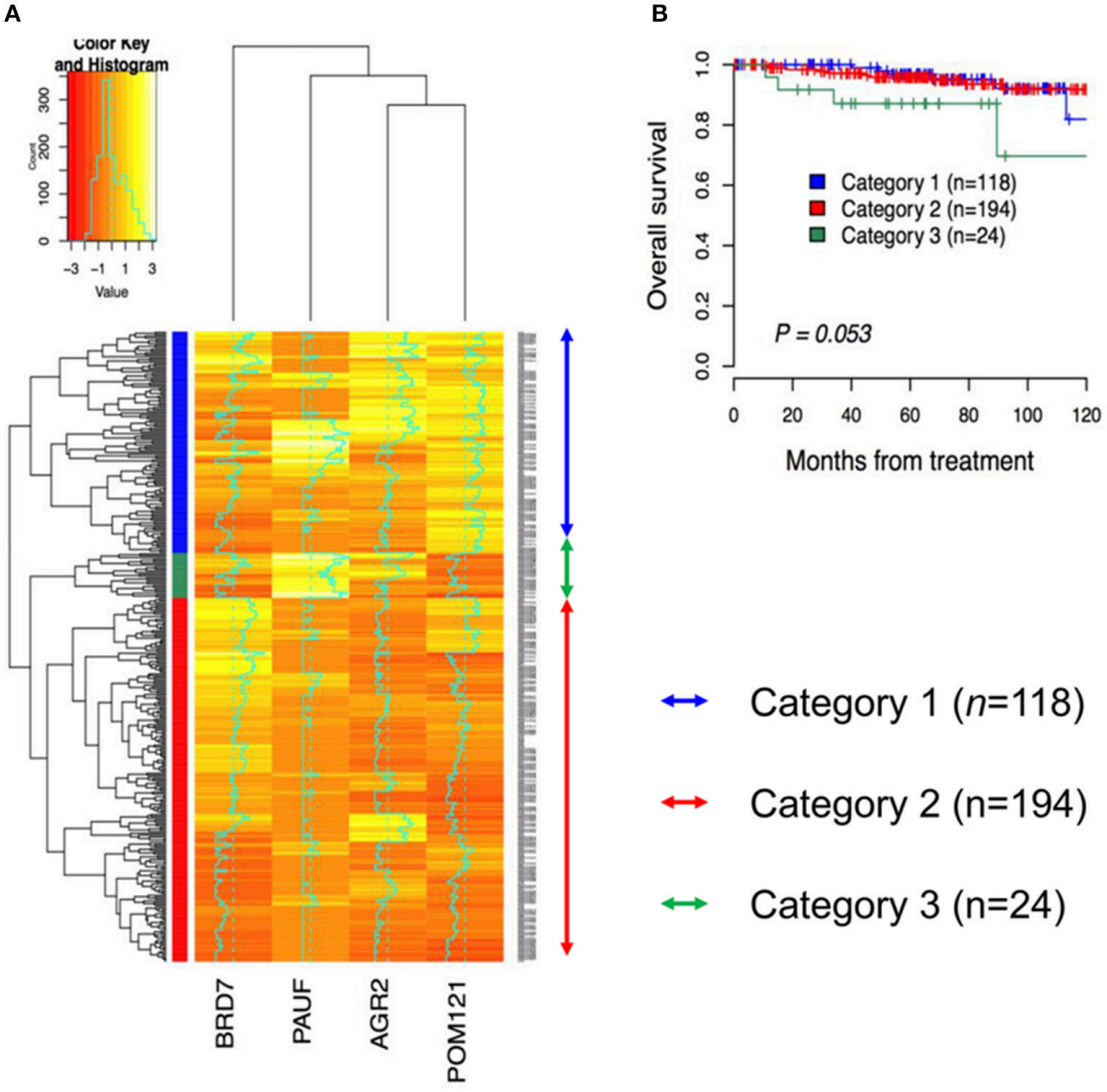
Hierarchical clustering analysis for immunohistochemical expression of PAUF-associated proteins in cervical cancer patients. **(A)** Three cluster groups were identified in the present study. Category 1 (*n* = 118, blue arrow) consists of relatively high AGR2 and POM121 expression. Category 2 (*n* = 194, red arrow) consists of relatively low AGR2 and PAUF expression. Category 3 (*n* = 24, green arrow) consists of relatively high PAUF and low POM121 expression. **(B)** Kaplan–Meier plot for overall survival according to group by hierarchical clustering. A tendency toward decreased overall survival was observed in Category 3 than in the other groups (*log*-rank test, *p* = 0.053).

To examine whether patients can be grouped according to protein expression, a total of 336 cervical cancer cases were analyzed using hierarchical clustering with histoscore. As shown in Figure 3A, the patients were divided to three groups. Category 1 (*n* = 118) consisted exclusively of AGR2^High^ and POM 121^High^ expression. Category 2 (*n* = 194) consisted exclusively of AGR2^Low^ and PAUF^Low^ expression. Category 3 (*n* = 24) consisted of PAUF^High^ and POM 121^Low^ expression. There were significant differences between categories 1, 2, and 3 in terms of cell type, depth of invasion, and radiation therapy resistance (*p* < 0.001, *p* = 0.008, and *p* < 0.001, respectively; Table 2). Notably, category 3 was associated with adenocarcinoma cell type and the radiation-resistant phenotype and showed a poor overall survival (OS) tendency (*p* = 0.053; Figure 3B).

**Table 2:** Clinicopathological characteristics between three groups defined with hierarchical cluster analysis.

	**Category 1 *n* (%)**	**Category 2 *n* (%)**	**Category 3 *n* (%)**	***p-*value**
**AGE**				
<50 years	71 (35)	118 (58)	14 (7)	0.970
>50 years	47 (35)	76 (57)	10 (8)	
**FIGO STAGE**				
≤ IIA	101 (35)	167 (57)	23 (8)	0.380
IB2 or IIB	17 (38)	27 (60)	1 (2)	
**CELL TYPE**				
SCC	83 (32)	162 (63)	11 (5)	<0.001^*^
AD/ASC	35 (44)	32 (40)	13 (16)	
**TUMOR SIZE**				
≤ 4 cm	88 (34)	147 (57)	21 (8)	0.390
>4 cm	30 (37)	47 (59)	3 (4)	
**LVSI**				
Negative	78 (39)	109 (54)	15 (7)	0.240
Positive	40 (30)	84 (63)	9 (7)	
**DEPTH OF INVASION**				
<1 cm	61 (39)	79 (50)	17 (11)	0.008^*^
>1 cm	57 (32)	115 (64)	7 (4)	
**LN METASTASIS**				
Negative	91 (35)	145 (57)	20 (8)	0.620
Positive	27 (34)	49 (61)	4 (5)	
**PM INVOLVEMENT**				
Negative	111 (36)	171 (56)	23 (8)	0.140
Positive	7 (23)	23 (74)	1 (3)	
**RT RESISTANT**				
Resistant	2 (10)	13 (65)	5 (25)	<0.001^*^
Sensitive	41 (36)	69 (61)	3 (3*)*	
**PRIMARY TREATMENT**				
OP only	60 (35)	96 (56)	15 (9)	0.510
OP + RT	24 (34)	45 (64)	1 (2)	
OP + CCRT	32 (36)	50 (55)	8 (9)	
Neoadjuvant	2 (40)	3 (60)	0	

FIGO, International Federation of Gynecology and Obstetrics; SCC, squamous cell carcinoma; AC, adenocarcinoma; ASC, adenosquamous cell carcinoma; LVSI, lymphovascular space invasion; LN, lymph node; PM, parametrium; OP, operation; RT, radiotherapy; CCRT, concurrent chemoradiation.

* *Significant at the level of p < 0.05*.

### Prognostic significance of AGR2, BRD7, and POM121

**Figure 4:**
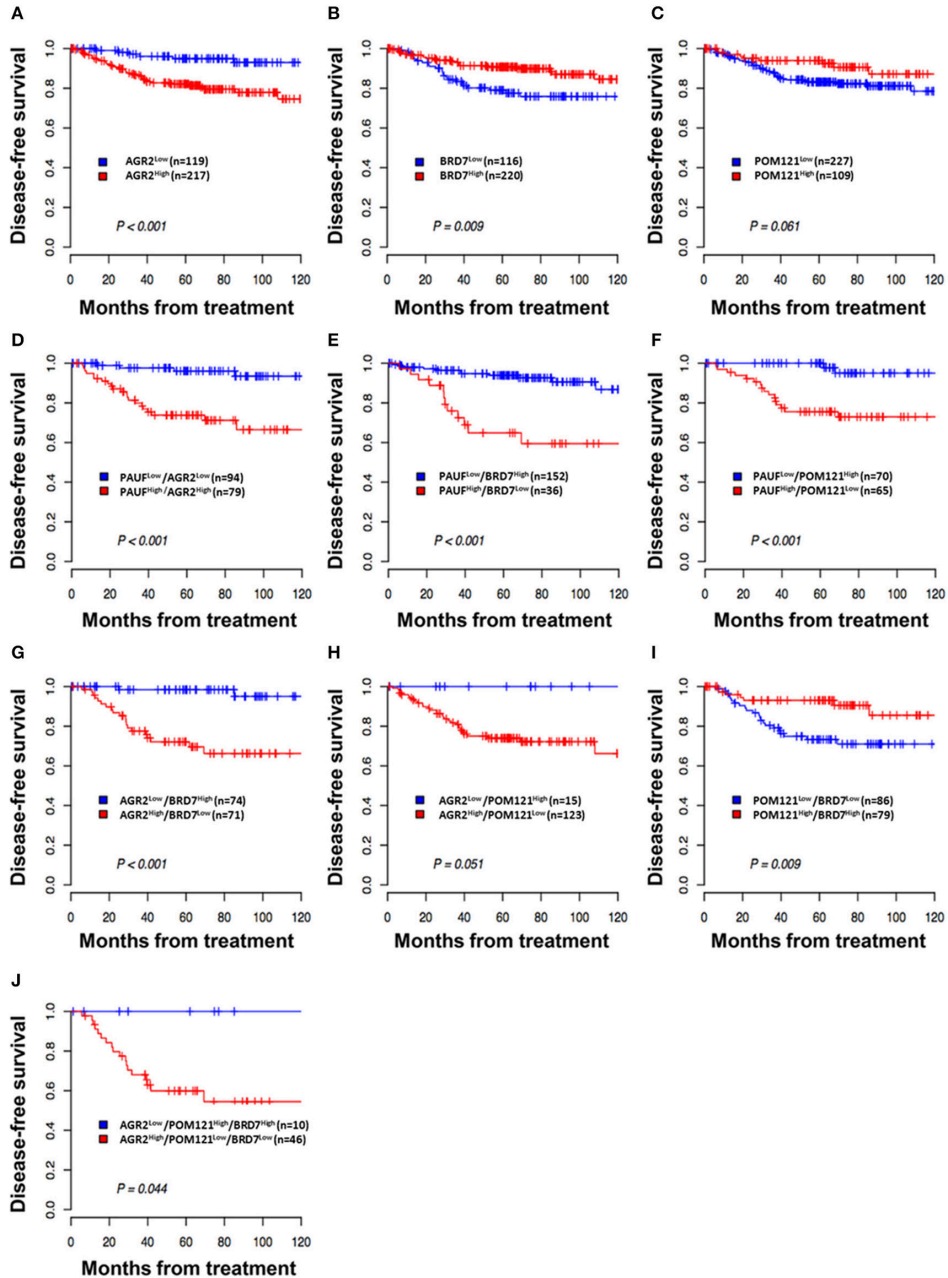
Kaplan–Meier plot of disease-free survival according to expression of PAUF and PAUF-associated proteins. **(A,B)** Patients with high AGR2 expression (AGR2^High^) and low BRD7 expression (BRD7^Low^) showed worse disease-free survival (*log*-rank test, *p* < 0.001 and *p* = 0.009, respectively) than patients with low AGR2 expression (AGR2^Low^) and high BRD7 expression (BRD7^High^). **(C)** Patients with low POM121 expression (POM121^Low^) showed a trend of worse disease-free survival. **(D–F)** Patients with combined PAUF^High^/AGR2^High^, PAUF^High^/BRD7^Low^, or PAUF^High^/POM121^Low^ expression showed shorter disease-free survival (*log*-rank test, *p* < 0.001, *p* < 0.001, and *p* < 0.001, respectively) than patients with the opposite combined expression profile. **(G–J)** Patients with combined AGR2^High^/BRD7^Low^, BRD7^Low^/POM121^Low^, or AGR2^High^/BRD7^Low^/POM121^Low^ expression showed shorter disease-free survival (*log*-rank test, *p* < 0.001, *p* = 0.009, and *p* = 0.044, respectively) than patients with combined AGR2^Low^/BRD7^High^, BRD7^High^/POM121^High^, or AGR2^Low^/BRD7^High^/POM121^High^ expression.

With a median follow-up period of 66 months (range 1–143), the 5-year disease-free survival (DFS) and OS rates for the whole group were 87% (95% CI 83–91) and 96% (95% CI 93–98), respectively. BRD7^High^ and AGR2^Low^ were significantly associated with favorable DFS (*p* = 0.009 and *p* < 0.001, respectively; Figures 4A,B). The 5-year DFS was 94.9% (95% CI, 90.7–99.4) for patients with AGR2^Low^ and 90.2% (95% CI, 86.2–94.4) and 94.0% (95% CI, 89.4–98.8) for patients with BRD7^High^ and POM121^High^ (Figure 4C), respectively, compared with 82.1% (95% CI, 76.9–87.7), 78.9 (95% CI, 71.1–87.7), and 83.2% (95% CI, 78.1–88.5) for patients with the opposite protein-expression pattern. Furthermore, we compared DFS using the combined expression of all three markers and PAUF according to a previous study (4). The combination of PAUF^Low^ with AGR2^Low^, BRD7^High^, and POM121^High^ had a significantly more favorable DFS than the opposite protein-expression profile (*p* < 0.001 for all; Figures 4D–F).

In addition, the combination of AGR2, BRD7, and POM121 expression had also significant prognostic significance. Patients with combined AGR2^High^/BRD7^Low^, BRD7^Low^/POM121^Low^, or AGR2^High^/BRD7^Low^/POM121^Low^ expression showed shorter disease-free survival (log-rank test, *p* < 0.001, *p* = 0.009, and *p* = 0.044, respectively) than patients with combined AGR2^Low^/BRD7^High^, BRD7^High^/POM121^High^, or AGR2^Low^/BRD7^High^/POM121^High^ expression (Figures 4G–J).

Similarly, the 5-year OS was 98% (95% CI, 95.2–100) for patients with AGR2^Low^ and 96.4% (95% CI, 93.9–99.1) and 97.0% (95% CI, 92.8–100) for patients with BRD7^High^ and POM121^High^, respectively, compared with 94% (95% CI, 90.7–97.6), 93.3% (95% CI, 88.3–98.7), and 93.9% (95% CI, 90.5–97.3) for patients with the opposite protein-expression profile (Figure S2). BRD7^High^ and AGR2^Low^ were significantly associated with favorable OS (*p* = 0.047 and *p* = 0.008, respectively; Figures S2A,B).

Using the Cox proportional hazards model, the expression of AGR2 and BRD7 remained independent prognostic factors for DFS (HR = 3.16; 95% CI, 1.32–7.58; *p* = 0.01 and HR = 0.50; 95% CI, 0.28–0.92; *p* = 0.025, respectively) in the multivariate analysis (Table 3). Furthermore, combined PAUF^High^ and AGR2^High^ showed independent prognostic value for both DFS and OS in the multivariate analysis (HR13.2; 95% CI, 3.72–46.89; *p* < 0.001 and HR43.07; 95% CI, 3.72–499.18; *p* = 0.003, respectively; Table S2).

**Table 3:** Univariate and multivariate analyses of disease free survival according to prognostic variables in cervical cancer patients (*n* = 336).

**Risk factor**	**Univariate**		**Multivariate**	
	**Hazard ratio [95%CI]**	***p-*value**	**Hazard ratio [95%CI]**	***p-*value**
FIGO stage (>IIB)	2.44 [1.24–4.82]	0.010^*^	2.29 [0.72–7.25]	0.160
Cell type (Adeno vs. SCC)	2.88 [1.62–5.14]	<0.001^*^	2.18 [0.75–6.28]	0.151
LN metastasis	4.13 [2.31–7.38]	<0.001^*^	4.52 [1.64–12.46]	0.004^*^
Tumor size (>4 cm)	1.7 [0.92–3.15]	0.092	0.65 [0.18–2.32]	0.508
PM involvement	2.24 [1.05–4.81]	0.038^*^	2.87 [0.85–9.66]	0.088
AGR2^High^	4.02 [1.7–9.49]	0.002^*^	3.16 [1.32–7.58]	0.010^*^
BRD7^High^	0.47 [0.26–0.84]	0.010^*^	0.5 [0.28–0.92]	0.025^*^
POM121^High^	0.5 [0.24–1.05]	0.066	0.52 [0.25–1.08]	0.078
PAUF^High^/AGR2^High^	7.17 [2.45–20.94]	<0.001^*^	13.2 [3.72–46.89]	<0.001^*^
PAUF^High^/BRD7^Low^	5.47 [2.4–12.48]	<0.001^*^	4.16 [1.57–11.02]	0.004^*^
PAUF^High^/POM121^Low^	9.21 [2.12–40.09]	0.003^*^	15.46 [2.68–89.08]	0.002^*^
AGR2^High^/BRD7^Low^	11.88 [2.77–50.95]	0.001^*^	8.15 [1.79–37.16]	0.007^*^
AGR2^High^/POM121^Low^	79227176.83 [0–Inf]	0.997	47108676.12 [0–Inf]	0.996
POM121^High^/BRD7^High^	0.34 [0.15–0.8]	0.013^*^	0.38 [0.16–0.91]	0.030^*^
AGR2^High^/POM121^Low^/BRD7^Low^	251011694.96 [0–Inf]	0.998	349083474.2 [0–Inf]	0.998

FIGO, International Federation of Gynecology and Obstetrics; SCC, squamous cell carcinoma; AC, adenocarcinoma; LN, lymph node; PM, parametrium.

* *Significant at the level of p < 0.05*.

### Improved prognostic power of combined molecular and clinical model

**Figure 5:**
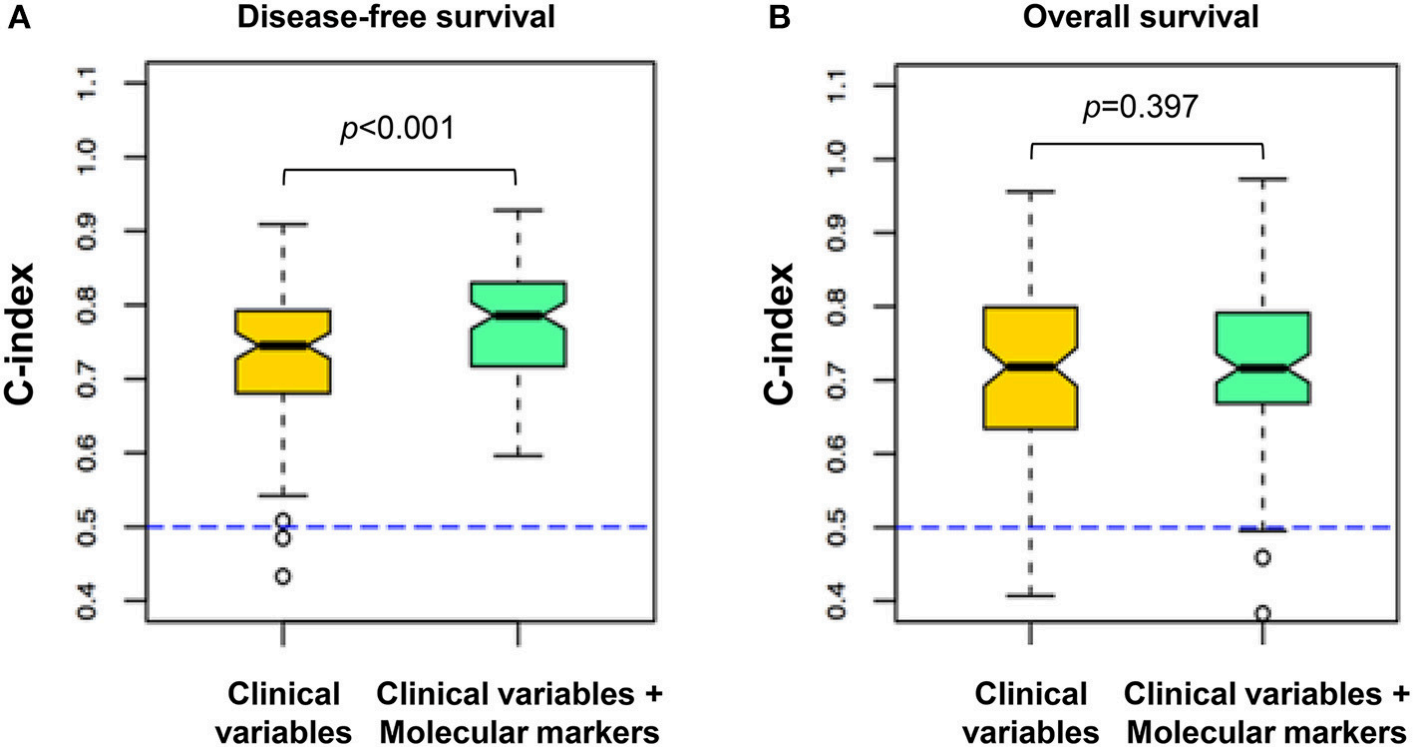
Comparison of survival predictive power between the clinical variables and the combined clinical and molecular data. The plots indicate the distribution of C-indexes from 100 rounds of cross-validation. The yellow box highlights the model built from the clinical variables, and the green box highlights the models integrating the molecular variables of PAUF-related proteins and the clinical variables. **(A)** Using the combined clinical and molecular markers (median C-index 0.79) to predict recurrence yielded improved predictive power compared with the clinical variable model (median C-index 0.74; *p* < 0.001). **(B)** The combined clinical/molecular-variable model showed similar performance compared with the clinical variable model in predicting death (*p* = 0.397). The dashed line indicates the C-index equivalent to a random guess (C-index = 0.5). A C-index of 1 indicates perfect prediction.

To examine whether a network-based model using expression of PAUF and related genes provided enhanced prognostic power compared to clinical prognostic factors (i.e., FIGO stage, lymph node metastasis, lymphovascular invasion, stromal depth of invasion, parametrial involvement, and resection margin), we compared the C-index for predicting recurrence of the clinical model and a combined clinical/network-based molecular model. In molecular model, PAUF and the related genes (AGR2, BRD7, and POM121) were included. Notably, the combined clinical/molecular model predicted recurrence (median C-index, 0.79; range, 0.55–0.93) with significantly higher power compared to the clinical variable-only model (median C-index, 0.74; range, 0.35–0.90; *p* < 0.001; Figure 5A), whereas, there was no difference in OS [C index of 0.71 (0.31–0.96) for combined model vs. 0.71 (0.33–0.94) for clinical model, *p* = 0.397; Figure 5B).

## Discussion

In the present study, we identified novel molecular markers interconnected with PAUF using genomic network-based analysis and evaluated their relationships and prognostic potential in a large cohort of cervical cancer patients. The identified proteins (AGR2, BRD7, and POM121) were differentially expressed according to cell type, clinical prognostic factors, and resistance to radiation therapy in cervical cancer patients. Furthermore, we demonstrated for the first time that low expression of AGR2, high expression of BRD7 or POM121 combined with low expression of PAUF predict delayed recurrence in cervical cancer patients. Based on these results, we suggest that a combined clinical/molecular variable model may have more prognostic potential than a model using clinical variables alone to predict disease recurrence. Based on the above findings, results from *in silico* analysis of publicly available genomic data were confirmed to show clinical significance with regard to protein levels of the new patient cohort in this study (11, 16).

The pro-oncogenic anterior gradient 2 (AGR2) protein stimulates cancer cell initiation, proliferation, invasion, and metastasis (17), and previous studies have shown that AGR2 is involved in p53 regulation and cell survival control (18). Additionally, elevated AGR2 protein expression is observed in numerous cancer types including gastric adenocarcinoma (19), breast (20), non-small cell lung (21), ovarian (22, 23), esophageal, prostate, and pancreatic cancer (24), and overexpression of the AGR2 protein is correlated with poor prognosis in these carcinomas. Monoclonal antibodies against AGR2 markedly reduce tumor growth and metastasis in pancreatic ductal adenocarcinoma, but this reduction has only been observed in preclinical mouse models (25). In gynecological cancer, overexpression of the AGR2 protein was reported in high-grade ovarian carcinoma, which is related with metastasis and poor prognosis (22, 23). Furthermore, AGR2 was associated with chemotherapy resistance in an analysis of survival in high-grade serous ovarian carcinoma (22). Consistent with previous studies, AGR2 expression was positively correlated with adenocarcinoma and poor prognosis in cervical cancer in this study.

It is well known that cervical cancers result from a persistent infection with high-risk types of HPV (26). HPV infects the basal cells of the squamous epithelium, causing entry of its DNA into the nucleus of the host cells. The HPV DNA replicates as the basal cells differentiate, and induce a dramatic increase in the expression of the two HPV oncoproteins (E6 and E7). Herfs et al. have suggested that cuboidal cells at the cervical squamocolumnar junction are the origin of cervical cancer and its precursors (27). They also identified the squamocolumnar junction cell-specific biomarkers keratin 7, AGR2, CD63, matrix metalloproteinase 7, and guanine deaminase and documented their expression in HPV-infected cell lines, high-grade cervical intraepithelial neoplasms, and cervical carcinoma (27). Recently, Morbini et al. showed that only AGR2 and keratin 7 were associated with active HPV infection in oropharyngeal carcinomas (28). Taken together, these data support AGR2 involvement in cervical cancer tumorigenesis by promoting the proliferation of human cervical cancer cells.

Bromodomain-containing protein 7 (BRD7), also known as celtix-1, is a subunit of the SWI/SNF complex specific for polybromo-associated BRG1-associated factor (PBAF), which was first identified as a tumor suppressor in nasopharyngeal carcinoma cells in 2000 (29). Published studies show that BRD7 plays an important role as a transcription co-factor in the expression of the TP53 gene (30) and interferes with the PI3K signaling pathways (31). Down-regulation of BRD7 expression has been observed in several malignancies and is correlated with poor prognosis of various cancer types, including breast (32), colorectal (33), gastric (29), and prostate cancer (34), as well as hepatocellular carcinoma (35), and osteosarcoma (36). Endometrial cancer (37) and ovarian cancer exhibit low expression of BRD7, which is correlated with down-regulated beta-catenin accumulation (38). In ovarian cancer, BRD7 expression is decreased in high-grade serous carcinoma relative to normal or low-grade carcinoma (38). In our study, high expression of BRD7 was associated with improved DFS in cervical cancer patients. Cox regression analysis confirmed that BRD7 and its combination with PAUF and other PAUF-related proteins is an important prognostic indicator in cervical cancer.

The nuclear envelope pore membrane protein (POM121) gene is one of the PAX5 fusion genes and is related to the development of B-ALL leukemia through gene rearrangement (39). POM121 is one of the integral membrane components of the nuclear pore complex (NPC) in vertebrate cells, which maintains NPC structure (40). Decreased POM121 expression significantly reduces assembled NPCs on the nuclear envelope and induces clustering of NPCs, which regulate molecular transport through the nuclear envelope (often referred to as the nuclear permeability barrier) (41). In our cervical cancer samples, low expression of POM121 alone was not correlated with decreased DFS. However, in combination with high expression of PAUF or low expression of BRD7, low expression of POM121 predicted significantly reduced DFS when compared with BRD7 or PAUF alone. BRD7 was found predominantly in the nucleus and the distribution ratio of BRD7 in nucleus/cytoplasm has effect on its function of transcriptional regulation (42). Also, in our previous study, PAUF had prognostic value when it was located in cytoplasm (4). Accordingly, we deduced that POM121 may have function of transporting BRD7 and PAUF proteins between nucleus and cytoplasm. However, further research is needed to identify the accurate mechanism of POM121 in cervix carcinoma tumorigenesis.

In this study, we performed integrative analysis using the cBioPortal to identify molecules in the PAUF pathway and found that AGR2, BRD7, and POM121 are possible important molecules of the oncogenic function of PAUF. Furthermore, we demonstrated that AGR2, BRD7, and POM121 protein expression had prognostic value when combined with PAUF expression using an analysis of a large cohort of cervical cancer patients. These findings suggest the usefulness of network-based prognostic markers because the prognostic accuracy of a clinical variable model was enhanced by the addition of molecular markers (i.e., AGR2, BRD7, and POM121). With these efforts to find novel molecular makers in cervical carcinoma prognostication, patients with high risk of recurrence might have opportunities to improve their treatment outcomes with closer follow up.

## Author contributions

JK, CC, J-YC, and SH conceived of the study and devised the experimental design. CC, J-YC, and JK performed the experiments. CC, J-YC, T-JK, J-WL, B-GK, D-SB, and SH performed data analysis for the experiments and the clinical records. CC, JK, and J-YC drafted the final version of the manuscript and figure legends. SH revised the figures, added critical content to the discussion, and was responsible for revising all submitted portions of the manuscript. All of the authors read and approved the final manuscript.

### Conflict of interest statement

The authors declare that the research was conducted in the absence of any commercial or financial relationships that could be construed as a potential conflict of interest.

## References

[1] Torre LA Bray F Siegel RL Ferlay J Lortet-Tieulent J Jemal A. Global cancer statistics, 2012. CA Cancer J Clin. (2015) 65:87–108. 10.3322/caac.21262 25651787

[2] Park JY Ngan HY Park W Cao Z Wu X Ju W. Asian Society of Gynecologic Oncology International Workshop 2014. J Gynecol Oncol. (2015) 26:68–74. 10.3802/jgo.2015.26.1.68 25609163 PMC4302288

[3] Koh WJ Greer BE Abu-Rustum NR Apte SM Campos SM Chan J. Cervical cancer. J Natl Compr Canc Netw. (2013) 11:320–43. 10.6004/jnccn.2013.0043 23486458

[4] Choi CH Chung JY Park HS Jun M Lee YY Kim BG. Pancreatic adenocarcinoma up-regulated factor expression is associated with disease-specific survival in cervical cancer patients. Hum Pathol. (2015) 46:884–93. 10.1016/j.humpath.2015.02.016 25870121 PMC7717069

[5] Hofree M Shen JP Carter H Gross A Ideker T. Network-based stratification of tumor mutations. Nat Methods (2013) 10:1108–15. 10.1038/nmeth.2651 24037242 PMC3866081

[6] Ideker T Krogan NJ. Differential network biology. Mol Syst Biol. (2012) 8:565. 10.1038/msb.2011.99 22252388 PMC3296360

[7] Cantor JR Sabatini DM. Cancer cell metabolism: one hallmark, many faces. Cancer Discov. (2012) 2:881–98. 10.1158/2159-8290.CD-12-0345 23009760 PMC3491070

[8] Hanahan D Weinberg RA. Hallmarks of cancer: the next generation. Cell (2011) 144:646–74. 10.1016/j.cell.2011.02.013 21376230

[9] Barabasi AL Gulbahce N Loscalzo J. Network medicine: a network-based approach to human disease. Nat Rev Genet. (2011) 12:56–68. 10.1038/nrg2918 21164525 PMC3140052

[10] Li J Lenferink AE Deng Y Collins C Cui Q Purisima EO. Identification of high-quality cancer prognostic markers and metastasis network modules. Nat Commun. (2010) 1:34. 10.1038/ncomms1033 20975711 PMC2972666

[11] Gao J Aksoy BA Dogrusoz U Dresdner G Gross B Sumer SO. Integrative analysis of complex cancer genomics and clinical profiles using the cBioPortal. Sci Signal (2013) 6:l1. 10.1126/scisignal.2004088 23550210 PMC4160307

[12] Choi CH Chung JY Chung EJ Sears JD Lee JW Bae DS. Prognostic significance of annexin A2 and annexin A4 expression in patients with cervical cancer. BMC Cancer (2016) 16:448. 10.1186/s12885-016-2459-y 27402115 PMC4940752

[13] Choi CH Chung JY Kim JH Kim BG Hewitt SM. Expression of fibroblast growth factor receptor family members is associated with prognosis in early stage cervical cancer patients. J Transl Med. (2016) 14:124. 10.1186/s12967-016-0874-0 27154171 PMC4859953

[14] Choi CH Chung JY Cho H Kitano H Chang E Ylaya K. Prognostic significance of AMP-dependent kinase alpha expression in cervical cancer. Pathobiology (2015) 82:203–11. 10.1159/000434726 26337566 PMC7722984

[15] Ishwaran H Kogalur UB. Consistency of random survival forests. Stat Probab Lett. (2010) 80:1056–64. 10.1016/j.spl.2010.02.020 20582150 PMC2889677

[16] Cerami E Gao J Dogrusoz U Gross BE Sumer SO Aksoy BA. The cBio cancer genomics portal: an open platform for exploring multidimensional cancer genomics data. Cancer Discov. (2012) 2:401–4. 10.1158/2159-8290.CD-12-0095 22588877 PMC3956037

[17] Brychtova V Vojtesek B Hrstka R. Anterior gradient 2: a novel player in tumor cell biology. Cancer Lett. (2011) 304:1–7. 10.1016/j.canlet.2010.12.023 21371820

[18] Pohler E Craig AL Cotton J Lawrie L Dillon JF Ross P. The Barrett's antigen anterior gradient-2 silences the p53 transcriptional response to DNA damage. Mol Cell Proteomics (2004) 3:534–47. 10.1074/mcp.M300089-MCP200 14967811

[19] Zhang J Jin Y Xu S Zheng J Zhang QI Wang Y. AGR2 is associated with gastric cancer progression and poor survival. Oncol Lett. (2016) 11:2075–83. 10.3892/ol.2016.4160 26998125 PMC4774612

[20] Barraclough DL Platt-Higgins A de Silva Rudland S Barraclough R Winstanley J West CR. The metastasis-associated anterior gradient 2 protein is correlated with poor survival of breast cancer patients. Am J Pathol. (2009) 175:1848–57. 10.2353/ajpath.2009.090246 19834055 PMC2774050

[21] Alavi M Mah V Maresh EL Bagryanova L Horvath S Chia D. High expression of AGR2 in lung cancer is predictive of poor survival. BMC Cancer (2015) 15:655. 10.1186/s12885-015-1658-2 26445321 PMC4596313

[22] Darb-Esfahani S Fritzsche F Kristiansen G Weichert W Sehouli J Braicu I. Anterior gradient protein 2 (AGR2) is an independent prognostic factor in ovarian high-grade serous carcinoma. Virchows Arch. (2012) 461:109–16. 10.1007/s00428-012-1273-4 22752467

[23] Sung HY Choi EN Lyu D Park AK Ju W Ahn JH. Aberrant hypomethylation-mediated AGR2 overexpression induces an aggressive phenotype in ovarian cancer cells. Oncol Rep. (2014) 32:815–20. 10.3892/or.2014.3243 24920423

[24] Dumartin L Whiteman HJ Weeks ME Hariharan D Dmitrovic B Iacobuzio-Donahue CA. AGR2 is a novel surface antigen that promotes the dissemination of pancreatic cancer cells through regulation of cathepsins B and D. Cancer Res. (2011) 71:7091–102. 10.1158/0008-5472.CAN-11-1367 21948970 PMC3541941

[25] Arumugam T Deng D Bover L Wang H Logsdon CD Ramachandran V. New blocking antibodies against novel AGR2-C4.4A pathway reduce growth and metastasis of pancreatic tumors and increase survival in mice. Mol Cancer Ther. (2015) 14:941–51. 10.1158/1535-7163.MCT-14-0470 25646014 PMC4710371

[26] Walboomers JM Jacobs MV Manos MM Bosch FX Kummer JA Shah KV. Human papillomavirus is a necessary cause of invasive cervical cancer worldwide. J Pathol (1999) 189:12–9. 10.1002/(SICI)1096-9896(199909)189:1<12::AID-PATH431>3.0.CO;2-F 10451482

[27] Herfs M Yamamoto Y Laury A Wang X Nucci MR McLaughlin-Drubin ME. A discrete population of squamocolumnar junction cells implicated in the pathogenesis of cervical cancer. Proc Natl Acad Sci USA. (2012) 109:10516–21. 10.1073/pnas.1202684109 22689991 PMC3387104

[28] Morbini P Capello GL Alberizzi P Benazzo M Paglino C Comoli P. Markers of squamocolumnar junction cells in normal tonsils and oropharyngeal cancer with and without HPV infection. Histol Histopathol. (2015) 30:833–9. 10.14670/HH-11-590 25644820

[29] Yu X Li Z Shen J. BRD7: a novel tumor suppressor gene in different cancers. Am J Transl Res. (2016) 8:742–8. Available online at: http://europepmc.org/abstract/MED/27158366 27158366 PMC4846923

[30] Drost J Mantovani F Tocco F Elkon R Comel A Holstege H. BRD7 is a candidate tumour suppressor gene required for p53 function. Nat Cell Biol. (2010) 12:380–9. 10.1038/ncb2038 20228809

[31] Chiu YH Lee JY Cantley LC. BRD7, a tumor suppressor, interacts with p85alpha and regulates PI3K activity. Mol Cell (2014) 54:193–202. 10.1016/j.molcel.2014.02.016 24657164 PMC4004185

[32] Harte MT O'Brien GJ Ryan NM Gorski JJ Savage KI Crawford NT. BRD7, a subunit of SWI/SNF complexes, binds directly to BRCA1 and regulates BRCA1-dependent transcription. Cancer Res. (2010) 70:2538–47. 10.1158/0008-5472.CAN-09-2089 20215511

[33] Wu WJ Hu KS Chen DL Zeng ZL Luo HY Wang F. Prognostic relevance of BRD7 expression in colorectal carcinoma. Eur J Clin Invest. (2013) 43:131–40. 10.1111/eci.12024 23215825

[34] Kikuchi M Okumura F Tsukiyama T Watanabe M Miyajima N Tanaka J. TRIM24 mediates ligand-dependent activation of androgen receptor and is repressed by a bromodomain-containing protein, BRD7, in prostate cancer cells. Biochim Biophys Acta (2009) 1793:1828–36. 10.1016/j.bbamcr.2009.11.001 19909775

[35] Chen CL Wang Y Pan QZ Tang Y Wang QJ Pan K. Bromodomain-containing protein 7 (BRD7) as a potential tumor suppressor in hepatocellular carcinoma. Oncotarget (2016) 7:16248–61. 10.18632/oncotarget.7637 26919247 PMC4941311

[36] Hu K Liao D Wu W Han AJ Shi HJ Wang F. Targeting the anaphase-promoting complex/cyclosome (APC/C)- bromodomain containing 7 (BRD7) pathway for human osteosarcoma. Oncotarget (2014) 5:3088–100. 10.18632/oncotarget.1816 24840027 PMC4102794

[37] Park YA Lee JW Choi JJ Jeon HK Cho Y Choi C. The interactions between MicroRNA-200c and BRD7 in endometrial carcinoma. Gynecol Oncol. (2012) 124:125–33. 10.1016/j.ygyno.2011.09.026 22015043

[38] Park YA Lee JW Kim HS Lee YY Kim TJ Choi CH. Tumor suppressive effects of bromodomain-containing protein 7 (BRD7) in epithelial ovarian carcinoma. Clin Cancer Res. (2014) 20:565–75. 10.1158/1078-0432.CCR-13-1271 24198243

[39] Nebral K Denk D Attarbaschi A Konig M Mann G Haas OA. Incidence and diversity of PAX5 fusion genes in childhood acute lymphoblastic leukemia. Leukemia (2009) 23:134–43. 10.1038/leu.2008.306 19020546

[40] Funakoshi T Maeshima K Yahata K Sugano S Imamoto F Imamoto N. Two distinct human POM121 genes: requirement for the formation of nuclear pore complexes. FEBS Lett. (2007) 581:4910–6. 10.1016/j.febslet.2007.09.021 17900573

[41] Talamas JA Hetzer MW. POM121 and Sun1 play a role in early steps of interphase NPC assembly. J Cell Biol. (2011) 194:27–37. 10.1083/jcb.201012154 21727197 PMC3135402

[42] Zhou M Liu H Xu X Zhou H Li X Peng C. Identification of nuclear localization signal that governs nuclear import of BRD7 and its essential roles in inhibiting cell cycle progression. J Cell Biochem. (2006) 98:920–30. 10.1002/jcb.20788 16475162

