# Supplementary material for: Genomic Network-Based Analysis Reveals Pancreatic Adenocarcinoma Up-Regulating Factor-Related Prognostic Markers in Cervical Carcinoma
Source: Front Oncol. 2018 Oct 23;8:465. doi: 10.3389/fonc.2018.00465 (PMC6206228; doi:10.3389/fonc.2018.00465)
Supplement: Supplementary file 1 [file Data_Sheet_1.docx]

***SUPPLEMENTARY MATERIAL***

**Genomic Network-Based Analysis Reveals Pancreatic Adenocarcinoma Up-Regulating Factor-Related Prognostic Markers in Cervical Carcinoma**

*Jihye Kim^1,^* ^†^*, Joon-Yong Chung^2,^* ^†^*, Tae-Joong Kim^1^, Jeong-Won Lee^1^,*

*Byoung-Gie Kim^1^, Duk-Soo Bae^1^, Chel Hun Choi^1,*^, Stephen M. Hewitt^2,**^*

*^1^Departments of Obstetrics and Gynecology, Samsung Medical Center, Sungkyunkwan University School of Medicine, Seoul 06351, Republic of Korea*

*^2^Experimental Pathology Laboratory, Laboratory of Pathology, Center for Cancer Research, National Cancer Institute, National Institutes of Health, Bethesda, MD 20892 USA*

^†^The first two authors contributed equally to this paper.

***Corresponding Author:** Chel Hun Choi, MD, Ph.D., Department of Obstetrics and Gynecology, Samsung Seoul Hospital, Sungkyunkwan University College of Medicine, 81 Irwon-ro, Gangnamgu, Seoul 06351, Korea. Fax: +82-2-3410-0630; Tel: +82-2-3410-3519; Email: [chelhun.choi@samsung.com](mailto:chelhun.choi@samsung.com)

****Corresponding Author:** Stephen M. Hewitt, MD, Ph.D., Laboratory of Pathology, Center for Cancer Research, National Cancer Institute, National Institutes of Health, MSC1500, Bethesda, MD 20892-1500, USA. Fax No.: +1-240-541-4470; Tel: +1-240-760-7171; Email: [genejock@helix.nih.gov](mailto:genejock@helix.nih.gov)

**Supplementary Table 1 |** The 21 genes expressed differentially according to ZG16B (the PAUF expression gene) expression (GSE44001, data obtained through GEO2R). Data are available at <http://www.ncbi.nlm.nih.gov/geo/query/acc.cgi?acc=GSE44001>. These results validate with mutual exclusivity analysis using TCGA cervix data.

| **Symbol** | **GSE44001** | | | |  | **TCGA_cervix**  **(mutual exclusivity)** | |
| --- | --- | --- | --- | --- | --- | --- | --- |
|  | ***log*FC¹** | **adj.*p*.val** | **t²** | **B³** |  | **Log OR⁴** | ***P* value *** |
| GCNT3 | 1.3567 | 0.003 | 5.37 | 5.7972 |  | 3.99 | 0.001 |
| C17orf28 | 0.7623 | 0.003 | 5.34 | 5.6674 |  | 1.45 | 0.032 |
| EPS8L3 | 1.002 | 0.02 | 4.85 | 3.8411 |  | 2.41 | 0.001 |
| AGR2 | 0.5631 | 0.02 | 4.82 | 3.7495 |  | 2.79 | <0.001 |
| MYO1A | 1.06 | 0.034 | 4.63 | 3.0789 |  | 3.24 | <0.001 |
| GPR98 | -0.683 | 0.034 | -4.62 | 3.0451 |  | <-0.3 | NS |
| GPRC5C | 0.5734 | 0.038 | 4.55 | 2.7996 |  | 1.88 | 0.004 |
| TCN1 | 1.1524 | 0.038 | 4.5 | 2.6184 |  | 2.13 | 0.047 |
| C9orf152 | 1.3397 | 0.038 | 4.49 | 2.5884 |  | 2.90 | <0.001 |
| LRRC31 | 1.1985 | 0.038 | 4.48 | 2.5475 |  | 0.43 | NS |
| FOXA3 | 1.243 | 0.038 | 4.46 | 2.4914 |  | 2.82 | <0.001 |
| SLC44A4 | 0.3701 | 0.038 | 4.44 | 2.4362 |  | 2.30 | <0.001 |
| PCDH24 | 0.4129 | 0.038 | 4.43 | 2.3945 |  | 2.37 | 0.009 |
| CATSPERB | 0.2538 | 0.038 | 4.41 | 2.3246 |  | 0.70 | NS |
| ERN2 | 1.1871 | 0.044 | 4.36 | 2.1686 |  | 2.90 | <0.001 |
| JAG1 | -0.5107 | 0.046 | -4.34 | 2.0764 |  | <-0.3 | NS |
| BCL2L15 | 0.2552 | 0.046 | 4.32 | 2.0196 |  | 3.27 | <0.001 |
| TM9SF1 | 0.3996 | 0.046 | 4.28 | 1.9017 |  | 0.25 | NS |
| HGD | 0.9749 | 0.046 | 4.28 | 1.9001 |  | 1.83 | 0.003 |
| MUPCDH | 0.5282 | 0.046 | 4.28 | 1.8953 |  | 2.88 | 0.004 |
| MUC13 | 0.9014 | 0.046 | 4.27 | 1.8652 |  | 0.73 | NS |

*Log* OR (odd ratio), Quantifies how strongly the presence or absence of alterations in gene A are associated with the presence or absence of alterations in gene B in the selected tumors.

¹ *log* FC

² t

³ B

⁴*Log* OR> 0: association towards co-occurrence; log OR ≤ 0: association towards mutual exclusivity;

*****Derived from Fisher Exact Test, significant means value < 0.05; marginal means value is 0.05–0.1.

**Supplementary Table 2 |** Univariate and multivariate analyses of overall survival according to prognostic variables in cervical cancer patients (*n* = 336).

| **Risk factor** | **Univariate** |  |  | | **Multivariate** | | | |  |
| --- | --- | --- | --- | --- | --- | --- | --- | --- | --- |
|  | **Hazard ratio [95%CI]** | ***p* value** | | |  | **Hazard ratio [95%CI]** | ***p* value** | | |
| FIGO stage (IIB) | 2.49 [0.9 - 6.87] | 0.078 | |  | 1.7 [0.59 - 4.87] | | | 0.325 |  |
| Cell type (Adeno *vs.* SCC) | 4.52 [1.85 - 11.06] | 0.001* | |  | 4.5 [1.77 - 11.43] | | | 0.002* |  |
| LN metastasis | 2.93 [1.22 - 7.06] | 0.017* | |  | 2.96 [1.15 - 7.6] | | | 0.024* |  |
| Tumor size (> 4 cm) | 1.48 [0.57 - 3.86] | 0.419 | |  | 1.02 [0.37 - 2.85] | | | 0.970 |  |
| PM involvement | 2.83 [0.94 - 8.48] | 0.064 | |  | 2.19 [0.67 - 7.15] | | | 0.196 |  |
| AGR2^High^ | 5.71 [1.32 - 24.67] | 0.020* | |  | 3.88 [0.86 - 17.4] | | | 0.077 |  |
| BRD7^High^ | 0.42 [0.17 - 1.01] | 0.054 | |  | 0.45 [0.18 - 1.12] | | | 0.087 |  |
| POM121^High^ | 0.39 [0.11 - 1.32] | 0.128 | |  | 0.42 [0.12 - 1.44] | | | 0.167 |  |
| PAUF^High^ /AGR2^High^ | 15.39 [1.94 - 121.88] | 0.010* | |  | 43.07 [3.72 - 499.18] | | | 0.003* |  |
| PAUF^High^ /BRD7^Low^ | 7 [1.97 - 24.92] | 0.003* | |  | 3.74 [0.89 - 15.67] | | | 0.071 |  |
| PAUF^High^ /POM121^Low^ | 641016439.31 [0 - Inf] | 0.998 | |  | 3000291202.76 [0 - Inf] | | | 0.999 |  |
| AGR2^High^ /BRD7^Low^ | 12.76 [1.63 - 100.12] | 0.015* | |  | 8.13 [0.97 - 68.43] | | | 0.054 |  |
| AGR2^High^ /POM121^Low^ | 78799957.96 [0 - Inf] | 0.998 | |  | 49525600.21 [0 - Inf] | | | 0.998 |  |
| POM121^High^ /BRD7^High^ | 0.22 [0.05 - 1.01] | 0.052 | |  | 0.2 [0.04 - 1.04] | | | 0.056 |  |
| AGR2^High^/POM121^Low^/BRD7^Low^ | 245784222.83 [0 - Inf] | 0.999 | |  | 199276524.83 [0 - Inf] | | | 0.999 |  |

Abbreviations: FIGO, International Federation of Gynecology and Obstetrics; SCC, squamous cell carcinoma; AC, adenocarcinoma; LN, lymph node; PM, parametrium.

* Significant at the level of *p* < 0.05.

**Supplementary Figure 1|** Expression of the PAUF-associated proteins AGR2, BRD7, and POM121 in patients with cervical cancer according to radiation treatment. (**A**) Correlations between PAUF and PAUF-related protein expression in radiation-sensitive cervical cancer tissues. PAUF and POM121 protein expression was positively correlated with AGR2 expression (*r* = 0.305; *p =* 0.001 and *r* = 0.456; *p* < 0.001, respectively). (**B**) Correlations between PAUF and PAUF-related protein expression in radiation-resistant cervical cancer tissues. BRD7 protein expression was positively correlated with POM121 expression (*r* = 0.550; *p =* 0.012).

**Supplementary Figure 2 |** Kaplan–Meier plots of overall survival categorized based on expression of PAUF and PAUF-associated proteins and their combination. (**A**-**B**) Patients with AGR2^High^ and BRD7^Low^ expression showed worse overall survival (*log*-rang test, *p* = 0.008 and *p* = 0.047, respectively) than patients with the opposite expression profile; however, POM21 expression did not have statistically significant effect on overall survival (**C**). (**D**-**G**) Patient with combined PAUF^High^/AGR2^High^, PAUF^high^/BRD7^low^, PAUF^High^/POM121^Low^, or AGR2^High^/BRD7^Low^ expression showed shorter overall survival (*log*-rank test, *p* < 0.001, *p* < 0.001, *p* = 0.003, and *p* = 0.002 respectively) than patients with combined PAUF^Low^/AGR2^Low^, PAUF^Low^/BRD7^High^, PAUF^Low^/POM121^High^, or AGR2^Low^/BRD7^High^expression.
