# Supplementary figures and images for: Genomic Network-Based Analysis Reveals Pancreatic Adenocarcinoma Up-Regulating Factor-Related Prognostic Markers in Cervical Carcinoma
Source: Front Oncol. 2018 Oct 23;8:465. doi: 10.3389/fonc.2018.00465 (PMC6206228; doi:10.3389/fonc.2018.00465)

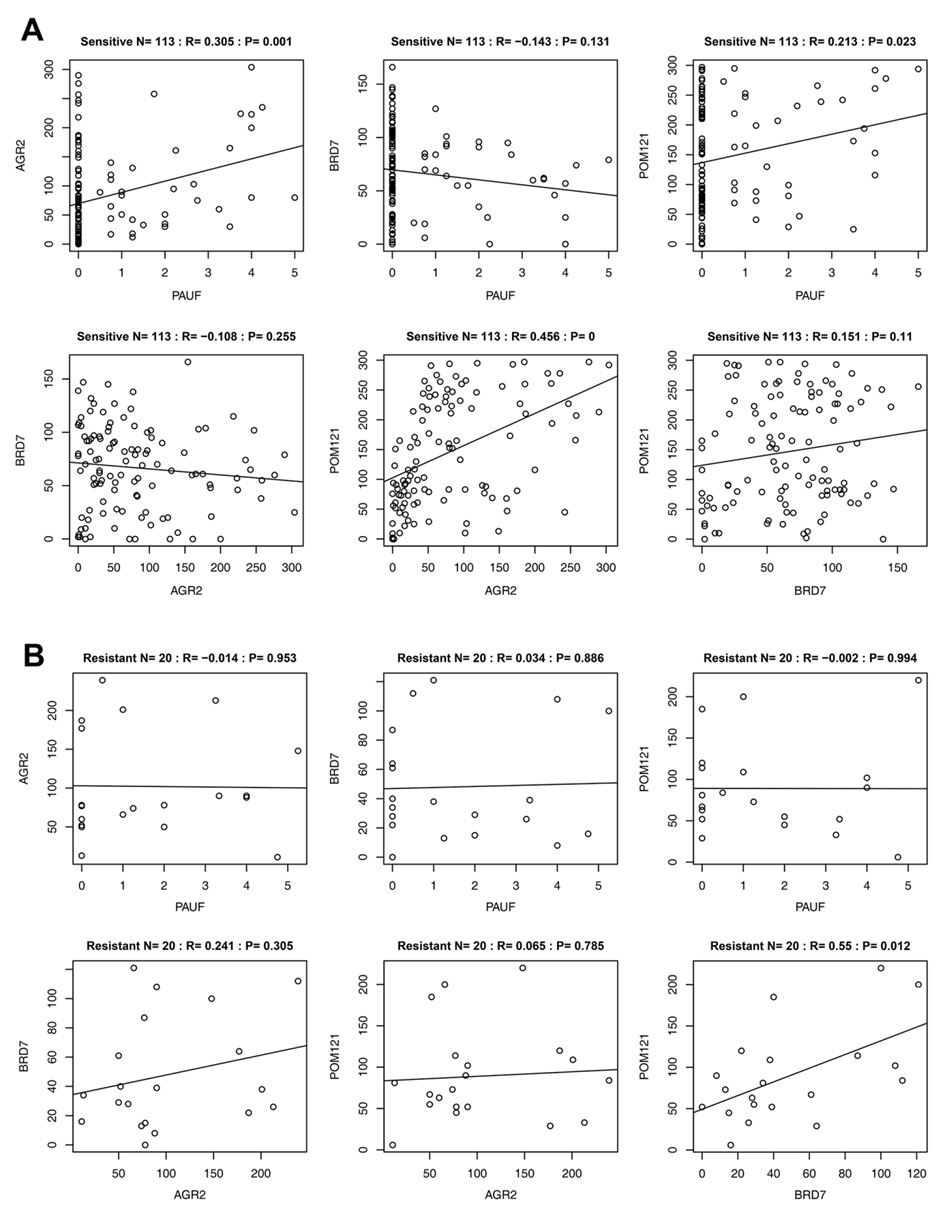

Supplement: Supplementary file 3 [file Image_1.TIF]

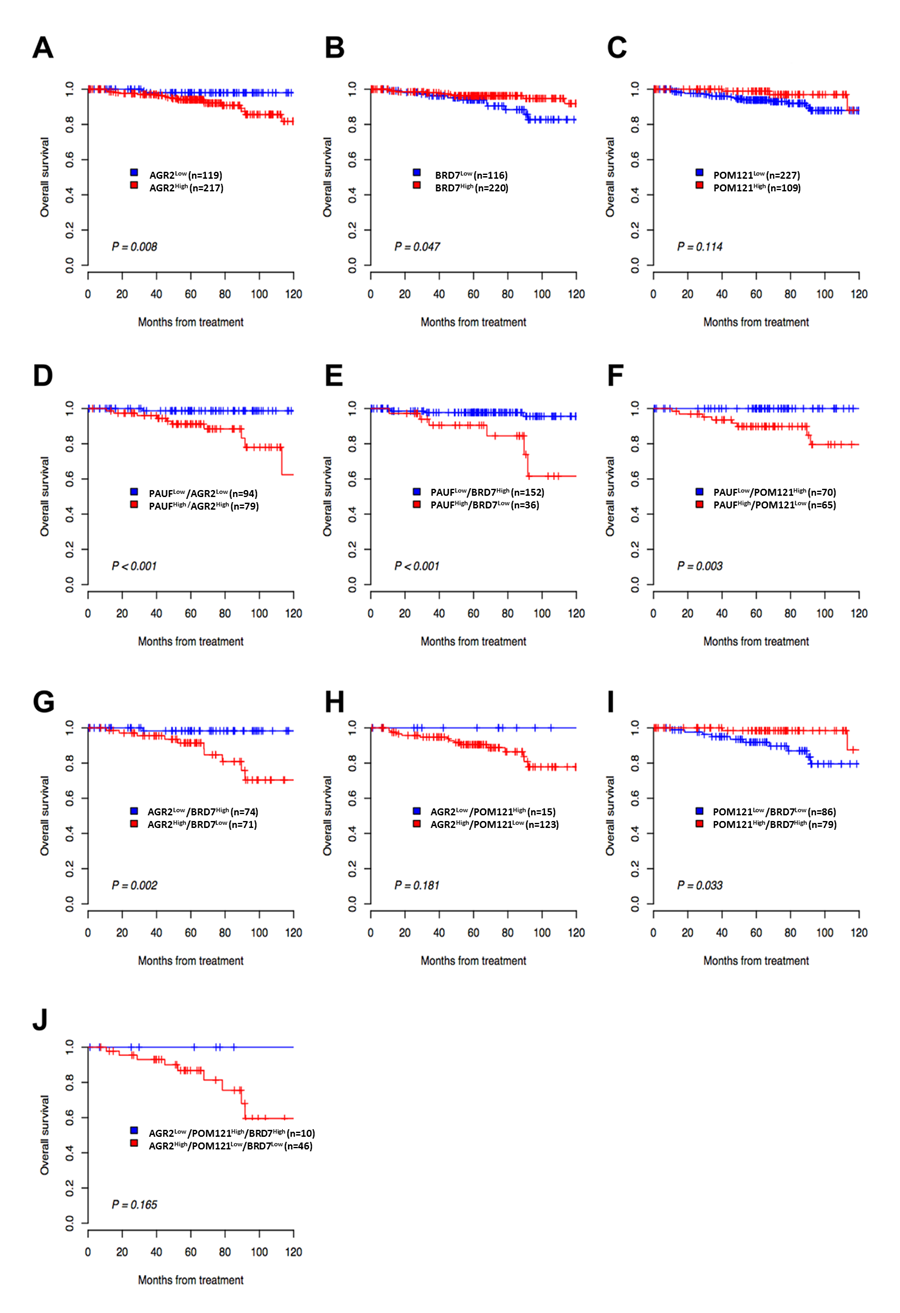

Supplement: Supplementary file 4 [file Image_2.TIF]
